# The impact of urban parks on the thermal environment of built-up areas and an optimization method

**DOI:** 10.1371/journal.pone.0318633

**Published:** 2025-03-06

**Authors:** Yujun Yang, Yuheng Lv, Dian Zhou

**Affiliations:** Department of Architecture, Xi’an Jiaotong University, Xi’an, Shaanxi, China; Changan University: Chang'an University, CHINA

## Abstract

Rapid urbanization, while transforming people’s living environments, also brings a series of urban issues such as the urban heat island effect. The urban park is an effective means to alleviate the urban heat island effect in summer. How to make better use of the cold island effect formed by urban parks to improve the urban outdoor thermal environment is an important topic. This manuscript takes Xi’an as the research area, using remote sensing data as the data source and combining field surveys, to explore the cooling characteristics of the cold island effect in the urban built-up area. It is demonstrated that, the influencing factors of the cooling effect of urban park cold islands are summarized: the area and perimeter of the park, the area and perimeter of water bodies, and the area of trees are all positively correlated with the cooling effect. The surrounding building density and building plot ratio are also positively correlated with the cooling effect of the green space. A comprehensive scoring model for each influencing factor is established, and the principal component analysis method is used to determine the weight of each indicator on the cooling effect of park design elements, among which the area of green space parks has the greatest influence weight. The demand space for cold islands in Xi’an’s parks is analyzed, and optimization strategies and suggestions for improving the urban thermal environment are put forward from both inside and outside the park.

## 1. Introduction

The urbanization rate of Chinese cities continues to rise and develop rapidly with construction and the rapid development of the economy, and this trend is expected to continue in the future. The rapid development of urbanization has brought about changes in the urban surface, with the increase in impermeable surfaces altering the rate at which the urban surface stores heat. An increasing amount of heat is stored on the surface, and coupled with the more frequent and diverse human activities in urban areas, this has led to an increasingly evident urban heat island effect. On a global scale, researchers in countries and regions at different stages of development and with different climatic environments have observed varying degrees of the heat island effect. The heat island effect has become a global phenomenon widely observed around the world. The existence and intensification of the heat island effect in urban areas have made high temperatures in summer a normalized phenomenon in cities. Studies have shown that the high temperatures in summer caused by the heat island effect have adverse effects on human health and has economic impacts in the form of energy waste.

In recent years, the focus of urban outdoor thermal environment research has been on how to address the negative impacts of urban heat island effects. The countermeasures proposed by existing studies mainly include two aspects: improving the urban spatial form and optimizing the types of underlying surfaces.

In terms of the urban spatial form, Oke first found a correlation between the shape of urban streets in the city center and the urban night-time heat island effect when studying urban and rural areas. The Sky View Factor (SVF), used to measure urban streets, is an important variable, and this variable is also considered to be the core of the relationship between urban scale and heat island intensity. Studies by Oke and other scholars report that the spatial form of the city has an important impact on the urban microclimate, laying the foundation for many subsequent studies on urban outdoor thermal environments [1]. A study conducted in Athens showed that a lower SVF value combined with denser green vegetation coverage can provide people with more livable bio-meteorological conditions [2]. There are also studies pointing out that the frequency of higher thermal comfort index increases with the increase in the SVF, and planting trees can significantly improve the SVF and reduce the heat of the outdoor thermal environment [3]. Many scholars have focused on the aspect of regional building density, and others believe that the density of urban construction areas has an important impact on the urban thermal environment. Brian states that the contribution of low-density residential development patterns to radiation heat energy is higher than that of high-density residential development patterns and suggests adopting a medium- to high-density building development model as a policy strategy to mitigate the impact of urban development on regional climate change [4].

In optimizing the types of underlying surfaces, researchers mainly focus on two factors: vegetation and water sources.

Vegetation mainly improves the outdoor thermal environment via a shading effect the transpiration of plant leaves [5]. Due to the shading effect of vegetation on sunlight, the shadow areas under the vegetation can significantly reduce the absorption of shortwave radiation, thereby reducing the surface temperature and air temperature. A study conducted in Hong Kong showed that planting trees in high-density environments can more effectively improve the thermal comfort of pedestrians than in open spaces [6]. In addition, trees with large canopies, short trunks, and dense foliage are most effective in reducing the average radiant temperature. The transpiration of plant leaves has been proven to effectively increase the surrounding air humidity, and the evaporation of water lowers the air temperature [7].

Regarding water sources, many scholars believe that water bodies improve the thermal environment mainly due to their reflectivity, high heat capacity, and large evaporation. After researchers studied the cooling effect of green space parks in Shenzhen, they found that the area of water bodies is one of the main factors affecting the cooling effect among many others [8]. Some researchers also state that water bodies can effectively promote air flow in the surrounding areas, thereby reducing air temperature. Experiments have shown that the air temperature near ponds has been significantly reduced by 2°C [9]. Some studies report that the evaporation cooling capacity of water bodies is an important measure in improving the urban outdoor thermal environment [10]. It is becoming increasingly important to effectively utilize the cooling effect of water bodies through appropriate landscape design and planning. Researchers conducted long-term fixed observations of rivers over 14 months, and the results showed that rivers brought about a reduction in air temperature of more than 5°C. However, this cooling effect is also affected by building density as well as wind speed and direction [11].

Studies have shown that green spaces in cities have a cooling island effect. Green cold islands within the built-up area often have lower internal temperatures than the surrounding areas, providing a comfortable thermal environment for residents entering them. Some researchers have pointed out that green cold islands can not only alleviate the negative feelings toward high urban temperatures for people entering them but also to some extent alleviate the heat island effect of the surrounding built-up areas.

In terms of the principle of cooling effects of green space, Francisco believes that this effect derives from the changes it makes to the underlying surface, as well as the changes in urban space reflectivity and solar radiation it triggers [12]. Ali suggests that the effect is caused by the shading effect of plants, with the shadows produced by plants in summer having a strong cooling effect on the ground [13], while Konarska states that it originates from the transpiration of plant leaves [14]. Some researchers have found that regardless of the form of streets and buildings, green space can reduce the temperature of the area [15].

Denise reports that the effect of vegetation on reducing air temperature is more pronounced in extreme high-temperature weather, with dense trees resulting in a temperature difference of 0.6°C in the block [16]. Sun found in his research that there is a negative correlation between impermeable surfaces and thermal comfort, and in various situations, green space can always improve the microclimate, improving thermal comfort for residents. Therefore, reducing the proportion of hard surfaces and increasing the proportion of green space is an effective way to improve thermal comfort [17,18].

Some researchers have tried to explore the factors behind spatial cooling. Hami believes that the types and form of buildings, their arrangement and combination, and the matching of trees and shrubs can affect the degree to which green space improves the environment [19]. Lee discusses the factors behind spatial cooling and finds that trees have a stronger effect in alleviating the urban heat island effect than grasslands [20]. Some researchers report that the temperature of green space is strongly related to its area. Wang, after using remote sensing technology to study Jilin City, stated that when the area of green space exceeds 50 hectares, the increase in its area leads to an effective reduction in its internal minimum temperature [21]. Perini compared ground greening with roof greening and reported that ground greening has a greater effect in reducing summer air temperature than roof greening [22]. Some researchers analyze the factors behind the green cold island effect from the perspective of wind. Morakinyo found in his research that improving the ventilation of the block can facilitate the cooling effect of green space in this area [15]. Lu discusses the cooling effect of large parks and, through software simulation, found that the intensity of a green cold island is closely related to wind speed. Excessive wind speed can lead to a reduction in the intensity of the green cold island, and changes in wind direction can also affect the lowest temperature center of the green park. The study shows that the impact of wind should be considered when designing green space [23].

The phenomenon where the temperature in green spaces or parks is lower than that of the surrounding built-up areas is commonly referred to as the urban green space cold island effect. An urban green space cold island is a type of urban cold island. The concept of the “cold island effect” in cities began to be studied after the emergence of the “heat island effect” and was developed on the basis of certain achievements in heat island effect research, while the study of urban green space cold island effects is based on the research of heat and cold island effects [18,24]. Studies have shown that urban parks and green spaces help to reduce high summer temperatures in cities and reduce summer cooling energy consumption [25].

The mechanism by which green space cold islands alleviate the heat island effect is different from other types of cold island effects in cities. The trees in green parks reduce solar radiation to the ground through their transpiration and shading effects, and water bodies lower the temperature within the green space through evaporation. The reflectivity of green spaces and water bodies to solar radiation is higher than that of artificially formed areas. Additionally, some studies suggest that the photosynthesis of plants can carry away some of the heat. The outdoor thermal environment in green parks is more comfortable under the influence of plant photosynthesis and the high heat capacity of water bodies. Moreover, studies have shown that urban green spaces, water bodies, and other landscapes not only provide cooler temperatures to people who enter them but also effectively alleviate the high temperatures in the surrounding built-up areas in summer, mitigating the impact of the heat island effect on people [26–28]. Masoodian found in his research that the intensity of a green cold island can reach 4.4°C, and the intensity of the cold island weakens in cold months and strengthens in warm months [24].

In terms of the green space cold island effect and its cooling patterns, after studying a large number of parks in Nagoya, Japan, Xin found that the size of the park, the area of shrubs and trees within the park, and a few other indicators have a decisive effect on the intensity of an urban green space cold island, while grasslands cannot enhance the cold island effect and have a negative impact on it [25]. Wang found in his research that green spaces smaller than 1.34 hectares do not have a green space cold island effect, the best park size is 1.3–17 hectares, and in park design, the underlying surface design should be considered, increasing vegetation coverage and water area while reducing lawn area and impermeable surfaces [29]. Wang believes that tree species richness and canopy cover are significantly positively correlated with the magnitude of temperature reduction, and the strength of their correlation varies with the season [30]. In summer, the greater the tree species richness and canopy cover, the greater the temperature reduction; in winter, the greater the tree species richness and canopy cover, the smaller the temperature reduction. In addition, improving tree species diversity also has a positive effect on increasing the intensity of the cold island. He states that the cooling effect of urban green spaces can be observed both in winter and summer, and the cooling effect in summer is stronger than in winter. In terms of the cooling distance of green parks, the research results show that the cooling distance of a green cold island in summer is roughly within a range of tens to hundreds of meters. Appropriately increasing the area or perimeter of urban green spaces can effectively enhance their cooling effect [31]. Chow, in his observation of the green space cold island effect in hot and arid areas, found that under the same conditions, the cooling distance of green parks varies in different directions, and its cooling effect is influenced by the layout of surrounding streets and buildings. The study also reports that the intensity of a green cold island is the greatest at its center, and the area near the geometric center of the park is generally the region with the lowest temperature. When comparing the bare ground and grassland in parks, Chow found that grassland in parks is cooler than bare ground, and areas with a higher SVF have lower cold island intensity [32]. Many scholars have also drawn conclusions regarding the influencing factors of urban green space cold island effects and the mechanisms behind them. Wu believes that the windward side of green spaces affects the intensity of the green cold island effect, and there is a positive correlation between them and the cooling distance of green cold islands [33].

To better apply this characteristic to practical projects, it is necessary to quantitatively analyze the cooling effect of urban green spaces. Arghavani explored the impact of different green scenarios in the Tehran metropolitan area on the early summer UHI [34]. Xu focused on the characteristics of park green spaces in the central urban area of Beijing and the differences in urban heat island effects across different spaces [35], and Wirayuda examined the relationship between urban green spaces and surface urban heat islands [36]. Rong, in his 2022 study, analyzed the spatiotemporal evolution of UHI in the central urban area of Xi’an and compared the cooling effects of green spaces and water in 13 city parks [37]. Imran categorized the characteristics of greening impacts within a day and found that green spaces can significantly reduce near-surface temperatures at night, but the effect is not obvious during the day [38].

Some scholars have applied the aforementioned research findings to actual planning. Han used a 500 m grid to analyze effects on pollutants and heat islands, finding significant mitigation of heat and PM pollution but increased O_3_ in summer. This suggests that planting low-BVOC trees and increasing green space can improve urban greening [39]. Liu proposed a new urban planning concept for Beijing to achieve a healthier environment through the spatial cooling effect of green spaces, clusters, and potential new communities [40]. Wang reveals that the cooling effects of urban green spaces vary with latitude and climate, with shape and NDVI impacts noted. Natural factors dominate in tropical and arid zones, while anthropogenic factors matter in temperate and continental zones, aiding urban planning [41]. Li’s research finds that optimal urban green space sizes and landscape metrics influence cooling, offering insights for urban heat mitigation in tropics [42].

In general, previous research on the green space cold island effect has focused on analyzing its causes and monitoring the intensity of the cold island, with little quantitative analysis. In terms of green space optimization strategies, most studies only provide qualitative suggestions, and those that can offer quantitative optimization strategies through research are even rarer. One main reason for this issue is that current research on the cooling effect of green spaces is concentrated on the macro scale, analyzing from the perspectives of solar radiation and the heat storage capacity of the underlying surface, with less research on specific quantitative analysis and optimization design for specific parks and green spaces. It is necessary to conduct quantitative research on the capacity of green spaces to improve the urban thermal environment to better understand the extent of the impact of urban green spaces on microclimate and urban heat island effects and to tap into the meteorological and environmental health potential of urban green spaces. The relevant conclusions can enable urban planners to adopt more scientific planning and design methods to optimize urban living environments and can also provide more quantitative design recommendations for the future construction of urban green spaces.

The convenience of remote sensing technology was leveraged in this study to conduct a quantitative analysis of the mechanisms affecting the green space cold island effect. Urban green space cold islands were taken as the research object, and the cooling characteristics of the urban green space cold island effect were explored. On this basis, the internal factors of green spaces (such as the scale of green spaces and the scale of water bodies) and external urban factors (such as the layout and form of surrounding buildings) that affect the green space cold island effect were explored, a quantitative model of the urban green space cooling effect and its internal and external comprehensive factors was established, and the magnitude and mechanism of their respective impacts were analyzed. The spatial demand for urban green space cold islands was also assessed, and ultimately targeted optimization and design strategies were developed for urban planners and policymakers. The research findings of this paper have implications for the urban planning of Xi’an and are also of great reference value for other cities with hot summers and cold winters.

## 2. Method

This study is based on Xi’an City as the research area and remote sensing data and Google Earth imagery data as the data source. The research method consists of analyzing the cooling effect of the urban green space cold island effect and the urban green space cooling effect with internal and external comprehensive factors and its influencing mechanisms by overlaying remote sensing image data with urban green space and urban land use data. The study was conducted as an extension of a project funded by the National Natural Science Foundation of China. The research design was reviewed and approved by the Expert Committee of the Institute of Human Settlements and Environment Science at Xi’an Jiaotong University, and opinions on community participation and behavior were provided before the commencement of the study. The modeling method used in this study was first proposed in a paper published by the research team in 2019 [43]. The core data collection for this study originated from April 2021, and the simulated results were compared again. The research conclusions and supporting data presented in this paper have been approved by all authors and their respective institutions.

### 2.1. Research area

Xi’an City is located in Shaanxi Province in the northwest of China (As shown in S1 Fig). Xi’an belongs to the cold region, so the research and planning construction of Xi’an City’s outdoor thermal environment has long focused more on the issue of keeping warm in winter, and there has been no in-depth study on the phenomenon of heat in summer. According to the meteorological data statistics in 2016, Xi’an City is the seventh-warmest provincial capital in China. It should be noted that the six cities ahead of Xi’an are all southern cities.

The reason for this characteristic is the topographical features of Xi’an and its surroundings. The terrain of the Xi’an urban area is mainly plains, but there are high mountains around, resulting in a topographical space similar to a “basin”. This topographical feature leads to a relatively high number of still wind days in the Xi’an urban area. Such wind environment characteristics make it difficult for the summer heat problem in Xi’an to be efficiently alleviated through the construction of urban-scale wind channels. Therefore, considering the characteristic of still wind days in Xi’an, the rational planning of green space has become an effective way to improve the summer thermal environment.

### 2.2. Data sources

The research data of this paper are divided into three aspects:

#### Surface temperature.

The surface temperature data are derived from the inversion of remote sensing data. The original remote sensing data used in this study were obtained from the digital products of the Landsat-8 series satellite from the United States. The remote sensing image map used was obtained from the website of the United States Geological Survey (http://www.usgs.gov). The Landsat-8 satellite image used was acquired at 10:55 AM on August 29, 2019, with a resolution of 30 m. It was sunny and cloudless that day, the image quality was good, and there was low image interference. Since the remote sensing satellite usually passes over the research area every 16 days, and the surface temperature inversion cannot be carried out if the remote sensing quality is poor or the cloud amount is large, there are very few remote sensing data available for the entire summer, which is one of the reasons the remote sensing data of this day were selected for the study.

#### Green space/park vector data.

The boundary information of green spaces, parks, and water bodies in the study area derives from the satellite remote sensing images of Google Earth. Vectorization operations were performed on these boundaries in the Arcgis software.

#### Building information data for the built-up area of Xi’an City.

The information of buildings and streets in Xi’an City, including building height, density, building plot ratio, etc., were obtained from the actual measurement data of the research team.

### 2.3. Mono-window algorithm

#### 2.3.1. Methods of computation.

The mono-window algorithm’s data foundation is derived from Landsat satellite imagery, and its derivation is based on the land surface thermal radiation transfer equation. According to the relevant literature, the error of this algorithm is between 0.4 and 1.1°C [44].

The calculation formula is as follows


$$\text{T=\{a×}\left(\text{1-C-D}\right)\text{+[b×}\left(\text{1-C-D}\right){\text{+C+D]×T}}_{\text{s}}{\text{-D×T}}_{\text{a}}\text{\}/C}$$ Formula 1

C=ε×τ Formula 2

$$\text{D=}\left(\text{1-τ}\right)\text{×[1+}\left(\text{1-ε}\right)\text{×τ]}$$ Formula 3

$${\text{T}}_{\text{a}}\text{=16.0110+0}{\text{.92621×T}}_{\text{0}}$$ Formula 4

where

T: The calculated land surface temperature;

a: Constant, with a value of −67.3553;

b: Constant, with a value of 0.4586;

Ts: Brightness temperature, in Kelvin;

*ε*: Land surface emissivity;

*τ*: Atmospheric transmittance;

T_0_: Air temperature at a height of 2 meters above the land surface, in Kelvin.

#### 2.3.2. Verification of calculation result.

The land surface temperature calculated with the single-window algorithm was used as the data source for this study. Our research team conducted a comparative validation between the remote sensing data inversion of the surface temperature and the actual measured surface temperature in Xi’an in 2019 in order to verify data accuracy and to prevent biases due to seasonal factors or differences in algorithms. Relevant studies have confirmed that the temperature inversion method employed by our research team is scientifically accurate [43].

**Fig 1:**
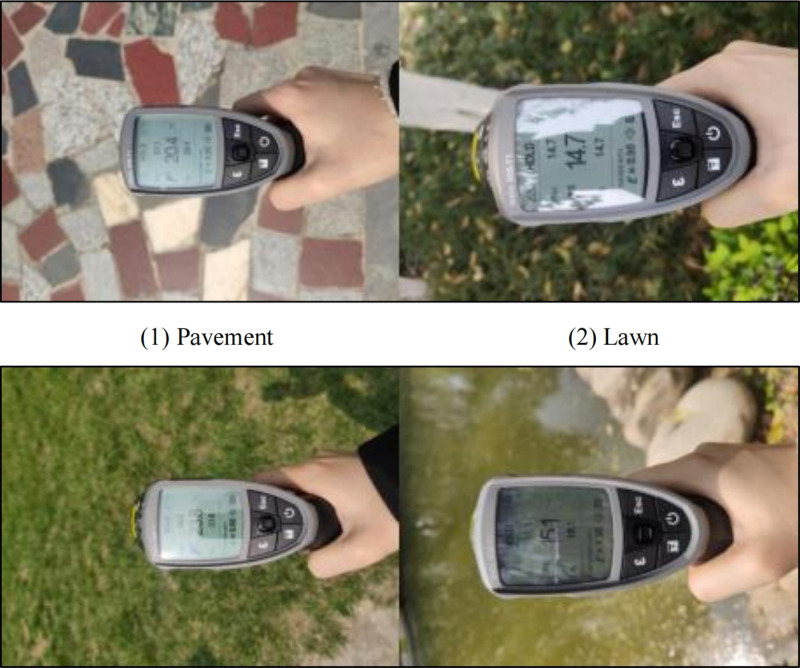
Measurement using Testo 835-T1 handheld infrared thermometer.

An actual land surface temperature measurement method was employed in this study to verify the accuracy of the land surface temperature retrieval. Verification was conducted using a Testo 835-T1 handheld infrared thermometer (Fig 1). The temperature range of this instrument is from −30°C to 600°C; the instrument’s accuracy is ± 0.1°C or ± 1%, whichever is greater; and the laser aiming method is a four-point laser targeting system. The measurement was conducted on the morning of April 12, 2021, from 10 to 11 AM, coinciding with the satellite’s overpass time. The weather in Xi’an City was clear and stable on that day. The test was carried out simultaneously at two test points within Xi’an City, which are Hancheng Lake Park and Wenjing Park. These two test points are also among the sample green parks in this study. The testing method involved using a handheld infrared thermometer to obtain surface temperature data from four types of underlying surfaces: pavements, lawns, areas under trees, and water surfaces. Each type of underlying surface was measured 20 times, resulting in a total of 160 sets of data.

**Fig 2:**
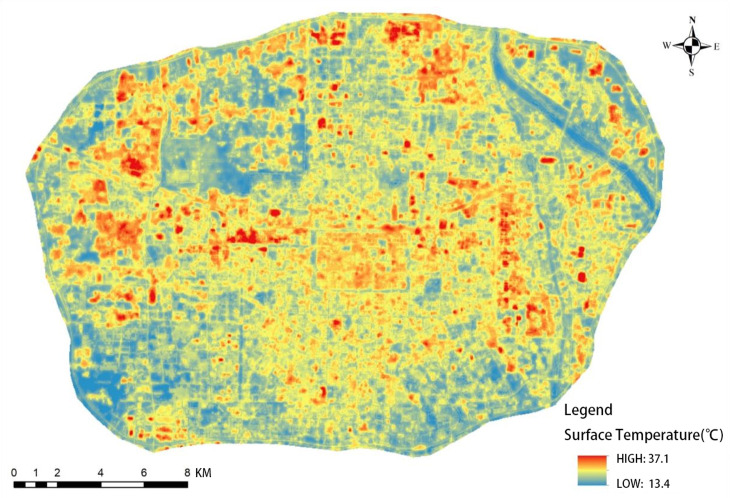
Inverse surface temperature result.

After obtaining the actual measurement data, land surface temperature retrieval calculation was performed on the remote sensing image of April 21, 2021, based on the aforementioned calculation method, and the results are shown in Fig 2.

**Fig 3:**
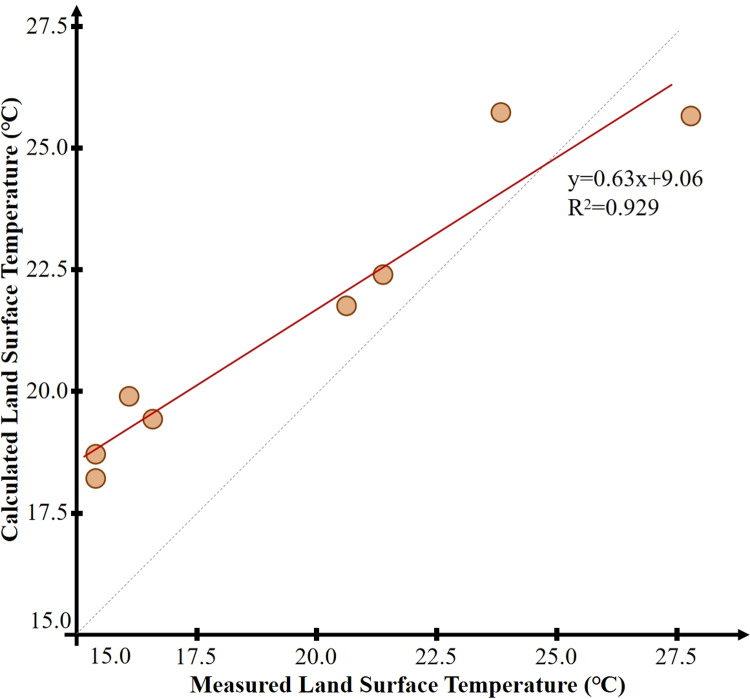
Fitting chart of difference between measured and calculated surface temperature.

An analysis was performed to determine the average land surface temperatures for the four types of underlying surfaces within the two parks. Through ArcGIS, random samples of the average surface temperatures for the four types of underlying surfaces were extracted for Hancheng Lake Park and Wenjing Park. A correlation analysis was conducted between the actual measured surface temperatures and the surface temperatures retrieved from the satellite data. The correlation coefficient was found to be 0.93, and the P-value was less than 0.01(Fig 3). It can be seen that the difference between the actual measured surface temperatures and the surface temperatures retrieved from the satellite data is relatively small. It can be concluded that the mono-window algorithm used in this study is capable of reflecting the true land surface temperatures. This conclusion is also consistent with the findings of similar studies [34].

### 2.4. Green space park details

In order to investigate the role of green space cold islands in mitigating urban heat island effects, it is essential to identify these areas within the city and select representative green parks through field surveys to assess their actual conditions. Low-temperature zones were identified in this study based on urban surface temperature data, excluding areas smaller than 0.01 km². Through on-site investigations, 36 green parks were selected as this study’s green cold island samples. We statistically analyzed the green space scale, presence of landscape lakes, and the proportion of tree canopy coverage. Because urban parks and green space construction in China must comply with the “Urban Green Space Classification Standard (CJJT85-2017)”, we chose the green space division method stipulated by the government to categorize the green spaces into four types: 1 for small (0–0.1 km²), 2 for medium (0.1–0.4 km²), 3 for large (0.4–0.7 km²), and 4 for extra-large (over 0.7 km²). Water features are divided into two categories: N for no water bodies and W for the presence of water bodies. The proportion of tree canopy coverage within the parks is categorized into four classes: Class 1 (0–20%), Class 2 (20%–40%), Class 3 (40%–60%), and Class 4 (over 60%). The research subjects are detailed in S1 Table.

## 3. Results

### 3.1. The impact of park design elements on the cold island effect

#### 3.1.1. Park perimeter.

**Fig 4:**
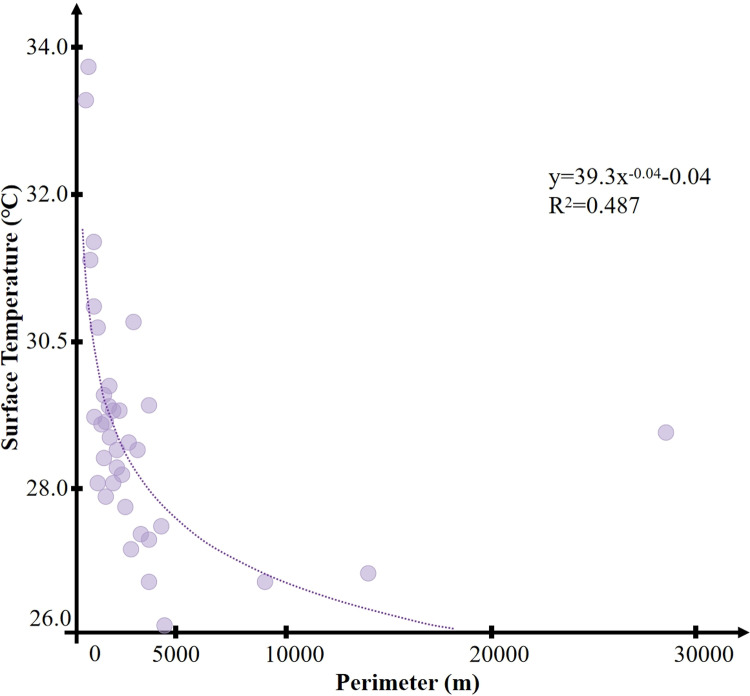
Correlation between park perimeter and temperature.

It was determined by calculating the correlation between the perimeter of the green space and its internal minimum temperature that the correlation coefficient is 0.487 (P <  0.01). It can be considered that there is a significant correlation between the perimeter of the green space and its internal minimum temperature. The park with the smallest perimeter is Park 5, with a minimum temperature of 33.26°C; the park with the largest perimeter is Park 33, with a minimum temperature of 28.72°C. As shown in Fig 4, in all the research samples, the majority of the parks’ perimeters are concentrated in the range of 0–4500 m, with only three parks having perimeters that are significantly larger than the other 33 cold island areas. These three parks are Parks 31, 33, and 36. The perimeter of Park 31 is 8952.42 m, with a minimum temperature of 26.7°C; Park 33 has the largest perimeter of 28424.19 m, with a minimum temperature of 28.72°C; and the perimeter of Park 36 is 13938.94 m, with a minimum temperature of 26.82°C. These three parks have exceptionally long perimeters and extensive spans due to their unique historical and archaeological status. For instance, Park 33 spans across three administrative districts in Xi’an City, with a diverse and complex surrounding environment. Additionally, parks with special archaeological or historical value like these will essentially be impossible to construct in newly built cities in the future.

**Fig 5:**
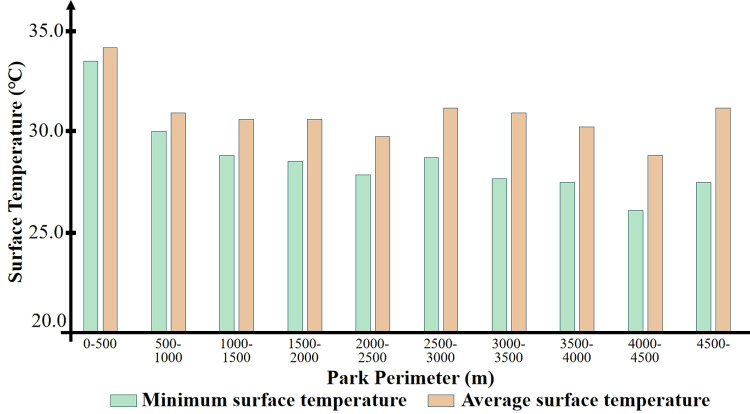
Park perimeter range and internal surface temperature.

The 36 parks were categorized based on their perimeters to more clearly determine the green space perimeter that can achieve the lowest internal temperature effect. All parks were divided into 10 intervals of 500 meters each (the typical length of the blocks in Xi’an is about 500 meters), and their internal average and minimum temperatures were statistically analyzed, resulting in the following cases: two parks in the 0–500 m category; six parks in the 500–1000 m category; seven parks in the 1000–1500 m category; seven parks in the 1500–2000 m category; three parks in the 2000–2500 m category; three parks in the 2500–3000 m category; three parks in the 3000–3500 m category; one park in the 3500–4000 m category; one park in the 4000–4500 m category; and three parks in the over 4500 m category. The statistical results of green cold island temperatures for each perimeter segment are shown in Fig 5. As illustrated, within the 4500 m perimeter, there is a clear trend of temperature decreasing with the increase in perimeter size. However, this trend is not evident in parks with a perimeter larger than 4500 m. This indicates that a larger perimeter of the cold island area does not necessarily result in a decrease in temperature. Once the park perimeter exceeds a certain range, further enlargement does not lead to a more significant cooling island effect.

#### 3.1.2. Park area.

**Fig 6:**
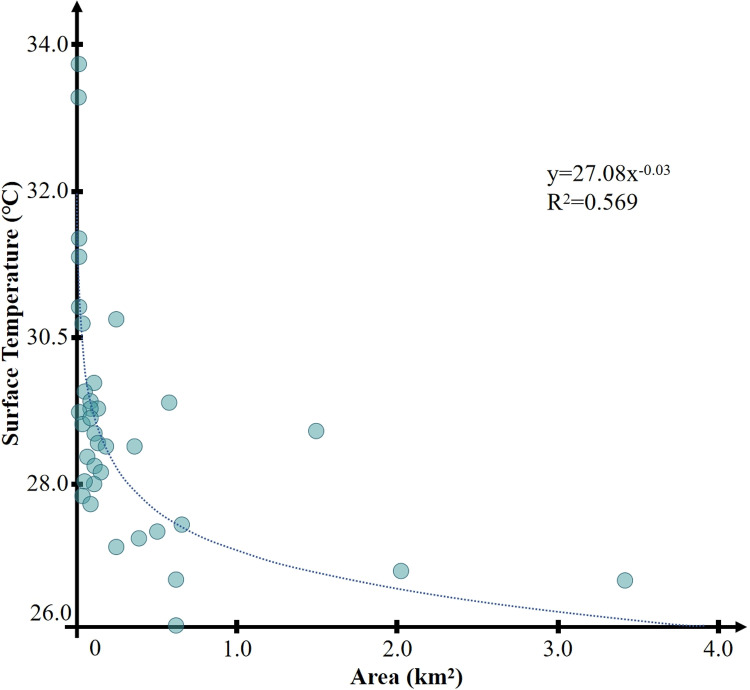
Correlation between park area and temperature.

Fig 6 describes the correlation between the area of green spaces and their internal minimum temperature, with an R^2^ of 0.569 and a P-value lower than 0.01, indicating a significant correlation. This suggests that there is a certain correlational relationship between the area of a park and its internal minimum temperature, and the two are negatively correlated. It should be noted that in all the statistical samples, the vast majority of parks have areas concentrated within the 0–0.7 km² range, with only three parks having areas significantly larger than the other 33, belonging to the category of extra-large parks. These three cold island areas are Parks 31 (3.4 km²), 33 (1.59 km²), and 36 (2.0 km²). The large area of these three parks does not result in a more pronounced low-temperature area, and the extremely large geometric areas of these three regions did not bring about a corresponding scale of temperature reduction. However, the lowest temperatures of all three were higher than those of Park 35, with an area of only 0.62 km². It can be considered that when the park area is within 0.7 km², there is a clear trend that the larger the area, the lower the minimum temperature inside the park. After the area exceeds 0.7 km², this characteristic is not obvious. Overall, as the area of the green space increases, the minimum temperature inside the park is lower, and the two show a more obvious negative correlation. As the park area gradually increases from 0.01 km², the minimum temperature in the green space area decreases rapidly with the increase in area, and when the area exceeds 0.7 km²; this change gradually tends to flatten. It should be pointed out that parks or green spaces larger than 0.7 km² are not common in cities, and they usually have special archaeological, historical, or ecological value.

**Fig 7:**
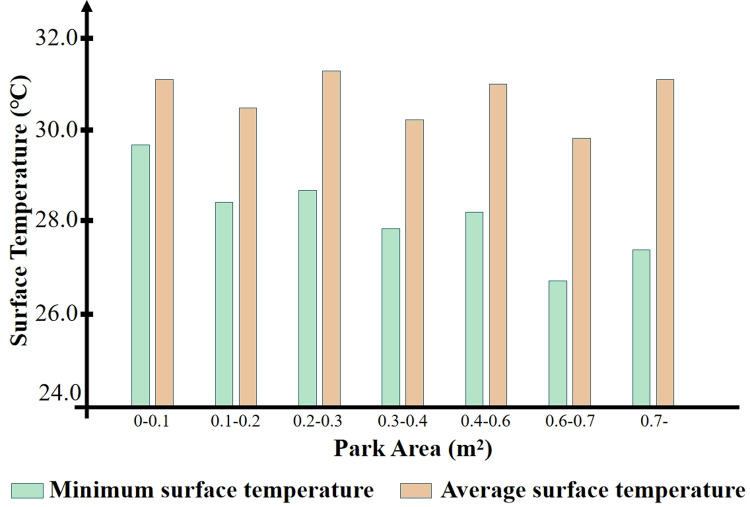
Park area range and internal surface temperature.

In actual city construction, it is not feasible to blindly build large green parks; instead, it is necessary to find the most scientifically effective range of park areas. In this study, 36 sample parks were categorized according to their size, dividing all parks into seven categories with an interval of 0.1 km², and statistics, namely, their internal average and minimum temperatures. Since there were no samples in the 0.4–0.5 km² range, it was merged into the 0.4–0.6 km² category. As shown in Fig 7, parks with an area within the range of 0.6–0.7 km² can provide the greatest cold island effect.

#### 3.1.3. Park shape index.

The Park Shape Index (PSI) of green space parks indicates the complexity of the park’s geometric form. The higher the shape index, the more complex the geometric form, the greater the contact area with the surrounding environment, and the more frequent the exchange of matter with the surrounding built environment. Therefore, the correlation between the shape index and the internal temperature was calculated in this study based on the calculation of the PSI for each park. The Pearson correlation coefficient between the two is low (R^2^ = 0.02, P < 0.01), and there is no correlation between them. Among the 36 parks, the one with the highest PSI is Park 33 (5.82), with a minimum temperature of 28.72°C; the one with the lowest PSI is Park 5 (0.94), with a minimum temperature of 33.26°C.

#### 3.1.4. Tree canopy coverage.

Existing studies have demonstrated that tall trees can reduce the surrounding thermal environment by shading shortwave radiation and through transpiration. In this study, the distribution of trees in 36 sample green parks was statistically surveyed, the area of arboreal trees in each park was calculated, and the proportion of arboreal area to the overall area of the green park was obtained to analyze the impact of arboreal trees on the internal temperature of parks based on remote sensing data and Google Earth combined with field investigations. The park with the largest arboreal area is Park 33, with an arboreal area of approximately 0.7683 km²; the park with the smallest arboreal area is Park 11, with an arboreal area of about 0.0006 km². The proportion of arboreal area in Xi’an’s parks ranges from 3.78% to 68.4%, with Park 1 having the highest proportion of arboreal area to the overall green park area (68.4%) and Park 31 having the lowest proportion (3.78%).

**Fig 8:**
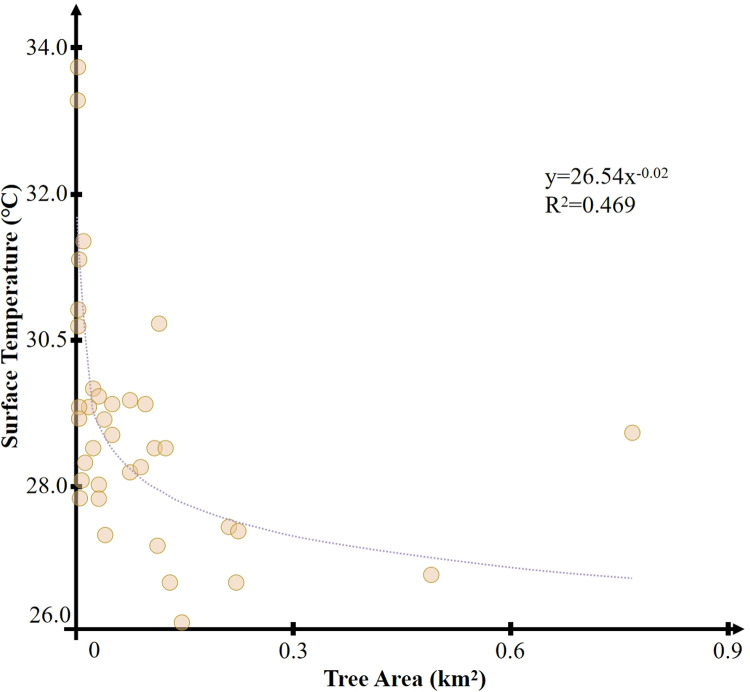
Correlation between tree area and temperature.

Fig 8 shows the correlation between the area of trees and the internal temperature of the park. The correlation between the two is 0.469, with a P-value lower than 0.01, indicating a significant correlation. This suggests that there is a notable negative correlation between the area of trees and the minimum internal temperature of green parks. The larger the area of trees, the lower the minimum internal temperature of the park. It should be noted that the correlation between tree cover ratio and the internal temperature of the park is extremely low (R^2^ = 0.0005). It can be inferred that planting more trees in larger parks can achieve a better cooling island effect, but in smaller parks, even with dense tree planting, the cooling effect of the park’s cooling island cannot be reliably ascertained.

#### 3.1.5. Water bodies.

Water bodies evaporate significantly during the day, which can reduce heat and have an important cooling effect on the surrounding environment. In the 36 parks studied in this work, 23 have water bodies, and 13 do not. In the parks with water bodies, the average area of the water body is 0.08 km². The smallest water body area appears in Park 14 (0.00071 km²), and the largest water body area appears in Park 36 (0.41 km²). The average water body perimeter is 3925.81 meters, the water body with the smallest perimeter appears in the Children’s Park as Park 14 (118.04 meters), and the water body with the largest perimeter appears in Park 33 (26122.74 meters). The average value of the water body shape index (WSI) is 3.05, the smallest shape index appears in Park 36 (0.25), and the largest water body shape index appears in Park 24 (12.63).

Comparing the internal temperature characteristics of all parks, it can be found that the average internal temperature of parks without water bodies is 31.6°C, while it is 30.5°C in parks with water bodies. The average minimum temperature of parks without water bodies is 30.0°C, while it is 28.1°C in parks with water bodies. The average and minimum temperature in parks with water bodies is generally lower.

**Fig 9:**
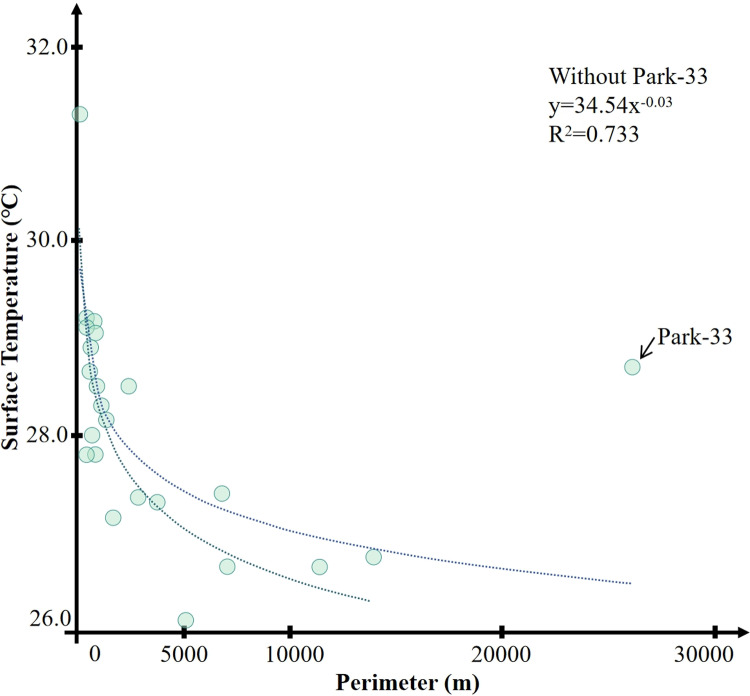
Correlation between water body perimeter and temperature.

Fig 9 shows the correlation between the perimeter of the water body and the minimum temperature of the park. The results show that the correlation is 0.500, P <  0.01, which is significant. This indicates that there is a strong correlation between the perimeter of the water body and the lowest internal temperature of the green space park. It should be pointed out that in all statistical samples, the vast majority of the park areas are concentrated within the range of 15000 meters, and only the perimeter of the water body in Park 33 (26122.74 meters) is much larger than that of the other parks. The R^2^ of the perimeters of the water bodies and the minimum temperature of the other parks is 0.733, P <  0.01.

**Fig 10:**
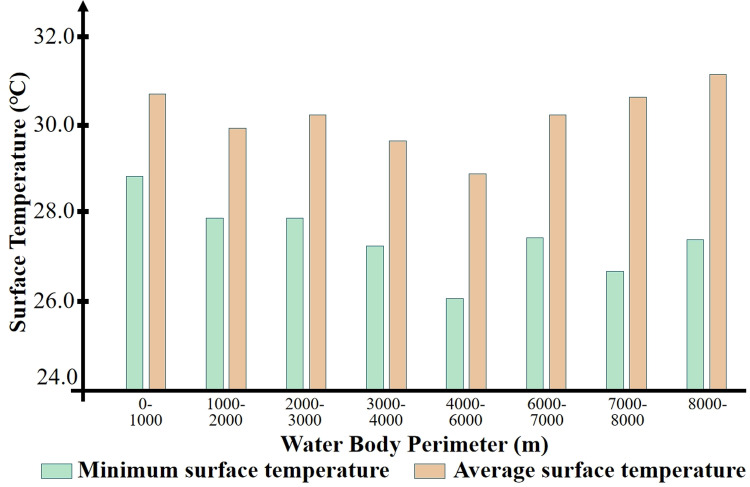
Water body perimeter range and internal surface temperature.

Analysis of the data reveals that when the perimeter of the water body is excessively large, the benefits of thermal environmental improvement brought about by the increase in perimeter are limited. We categorized the 23 sample parks according to their water body perimeters, dividing all parks into eight groups with intervals of 1000 meters, and statistically analyzed their internal average and minimum temperatures. As shown in Fig 10, when the perimeter of the water body within the park is within the range of 4000–6000 meters, the temperature is at its lowest, and the park’s cold island can reach the lowest internal temperature.

**Fig 11:**
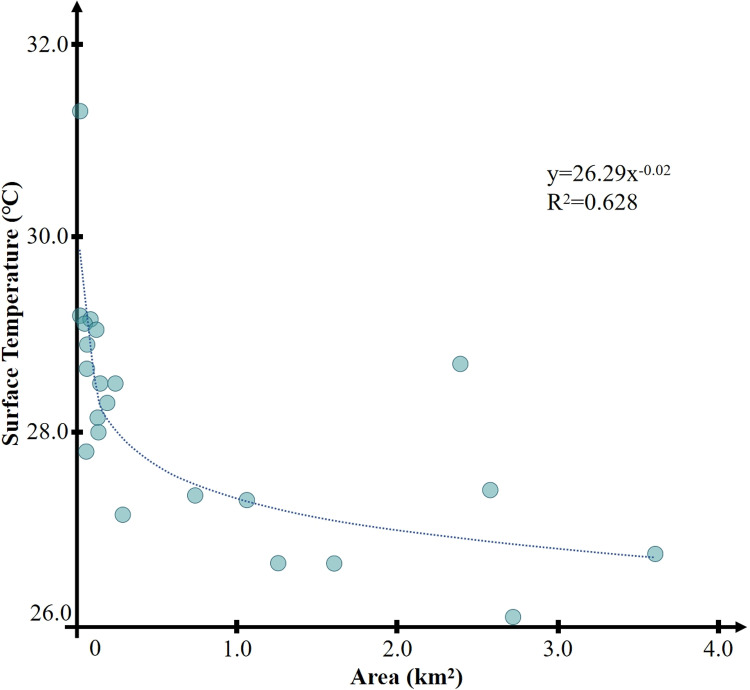
Correlation between water area and temperature.

Fig 11 shows the correlation between the area of the water body and the minimum temperature of the park. The results show that the correlation is 0.63, P <  0.01, which is significant. This indicates that there is a strong correlation between the perimeter of the water body and the lowest internal temperature of the green space park.

Similar to the perimeter, when the area of the water body is excessively large, the benefits of thermal environmental improvement brought about by the increase in area are limited. Moreover, in actual engineering projects, it is not feasible to blindly construct parks with large areas of water bodies. Therefore, the water body areas of 23 sample parks were analyzed in this study, and all parks were categorized into different groups with intervals of 0.1 km². The analysis results show that when the area of the water body within the park is within the range of 0.3–0.4 km², the green space cold island can reach the lowest internal minimum temperature and the lowest average temperature.

The water body shape index (WSI) within the park indicates the complexity of the geometric form of the water body in that area. The larger the WSI, the more complex the geometric form of the water body, the greater the contact area with the surrounding green space, and the more frequent the material exchange with the surrounding environment. Therefore, the water body shape index may have a certain impact on the internal temperature. However, after calculating the WSIs of 23 cases, it was found that the correlation coefficient between the shape index and the internal temperature is low (R^2^ = 0.02), indicating no correlation between the two. Among the 23 cold island areas, Park 24 has the highest WSI, with a value of 12.63, and the lowest temperature is 28.0°C; Park 36 has the lowest WSI, with a value of 0.25, and the lowest temperature is 26.8°C.

### 3.2. The impact of park surrounding characteristics on the cold island effect

The previous section’s analysis focused on the impact of the park’s design elements on the cooling effect. However, the layout of the built-up areas surrounding the park, namely, the density, height, and building plot ratio of the buildings, affect the movement and diffusion process of cold and hot air within them. Cold air from the park’s cold island encounters obstructions from buildings with different layout forms, especially when it diffuses toward the surrounding built-up areas, which changes its diffusion capability and ultimately affects the cooling effect of the green space cold island. Therefore, we analyzed the impact of the built-up area’s architectural layout form on the cooling effect of the park’s cold island.

#### 3.2.1. Surrounding building density.

Based on the statistical analysis of the building density characteristics of Xi’an City previously conducted by our research lab, we found that the city clearly exhibits a “centripetal” characteristic, meaning that the building density is higher in the city center and lower around the edges. The building density distribution map is detailed in S2 Fig. The area with the highest building density is located in the city center. This area has a long history of construction, with low building floors, close spacing between buildings, and mostly narrow streets. There are also two areas with relatively high building density in the northeast and southwest of Xi’an, which have been the main development zones of Xi’an City in recent years. The surface temperature retrieved through satellite inversion can provide the extent of the park’s cold island effect. As the spatial distance from the park increases, the change in surface temperature rises and gradually becomes gentler. The difference between the average temperature at the turning point and the average temperature within the green space was defined as the cooling extent (PCI) in this study. The concept of average cooling extent proposed by Hayder was adopted in the calculation method [45].

**Fig 12:**
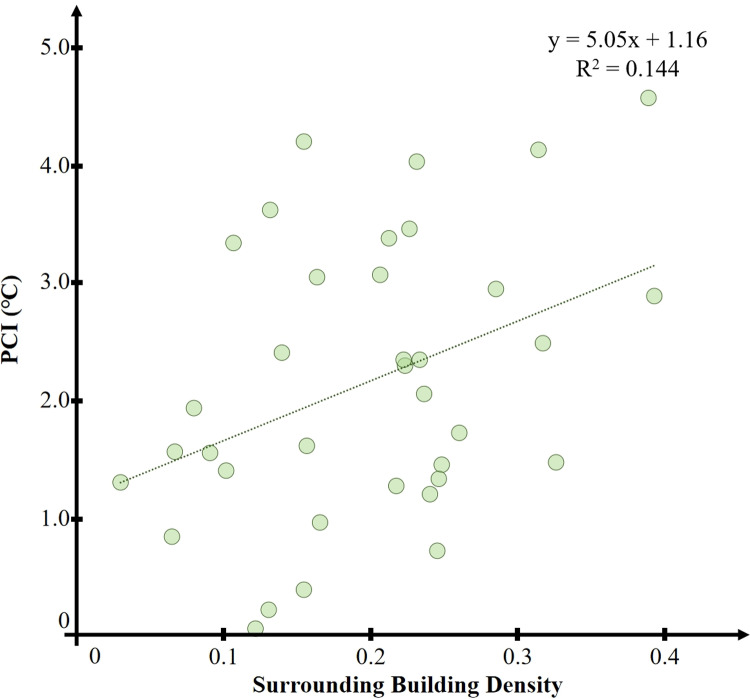
Correlation between park surrounding building density and PCI.

As shown in Fig 12, there is a general trend that the average cooling extent of the park’s cold island effect increases with the increase in the surrounding building density. However, the correlation between the two is relatively low, with an R^2^ =  0.14 and P <  0.01, indicating a statistically significant but weak correlation.

**Fig 13:**
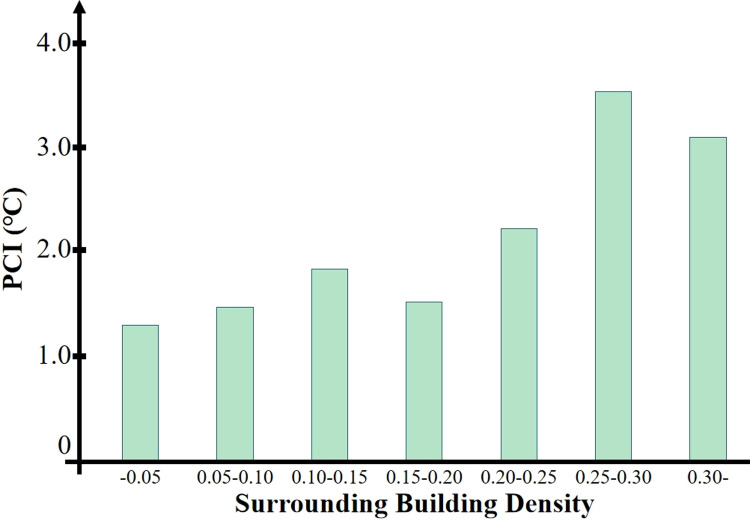
The impact of building density on the average cooling extent.

We categorized all parks into seven groups with an interval of 0.05 for building density and then statistically analyzed the cooling extent of each category to obtain the results shown in Fig 13. It can be observed that when the surrounding building density is above 0.25, the cooling extent of the park’s cold island effect is the greatest.

#### 3.2.2. Surrounding building plot ratio.

The building density can reveal the impact of urban layout in the horizontal direction on the cooling effect of parks, and the distribution characteristics of buildings in the vertical direction also affects the diffusion of cold air. The building plot ratio can describe the urban spatial information well from both horizontal and vertical perspectives. The size of the building plot ratio can reflect the intensity of land use, which not only covers the information of the building’s ground area but also the information of the building’s height. The distribution map of urban building plot ratios can be found in S3 Fig.

**Fig 14:**
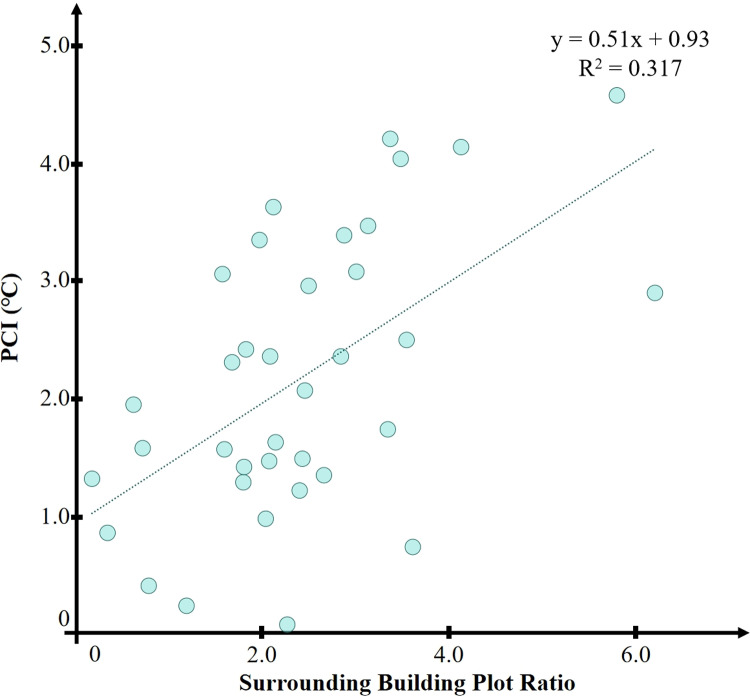
Correlation between park surrounding building plot ratio and PCI.

Fig 14 shows the correlation between the building plot ratio of the park’s surrounding area and the PCI effect. It can be observed that the correlation is relatively weak, with an R-squared value of 0.317 and a P-value lower than 0.01. However, the correlation is significantly stronger than that between the park’s surrounding building density and the PCI effect.

**Fig 15:**
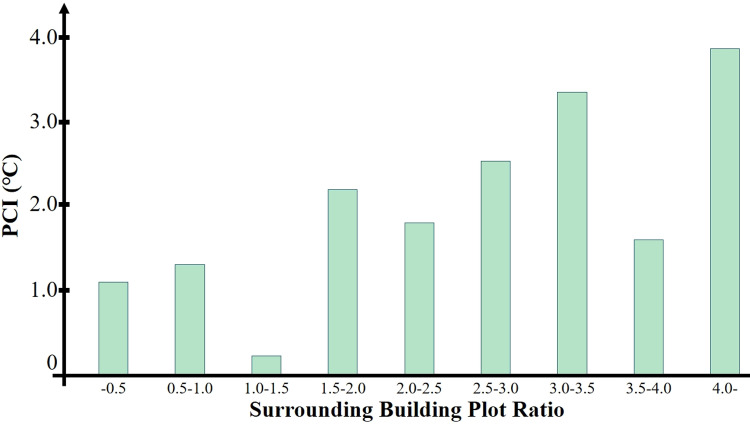
The impact of building plot ratio on the average cooling extent.

As shown in Fig 15, the building plot ratios of the 36 parks studied roughly range from 0 to 6. All sample parks were categorized into three levels based on a building plot ratio of 0.5. Analyzing the data shows that the green spaces with a building plot ratio above 4.0 in the surrounding construction area have the greatest cooling extent of the urban heat island effect, and the lowest cooling extent is within the range of a building plot ratio below 1.5.

## 4. Discussion

### 4.1. Weight analysis of influencing factors

Section 3 analyzes the various factors that affect the cooling effect of green space parks and their correlation sizes. To further guide planning and policy formulation, it is necessary to quantify and determine the impact weight of each element. Multiple linear regression analysis was employed to conduct a comprehensive analysis of each element index and to establish a comprehensive scoring model. Principal component analysis was used to couple the cooling effect with various influencing factors for analysis in order to determine the impact weight of each element on the park’s cooling effect. Based on the conclusions of previous research, seven main factors affecting the cooling effect of the park were identified: the park area, park perimeter, water area, water perimeter, proportion of tree canopy area, surrounding building density, and surrounding building plot ratio.

Since the magnitude of each indicator is different, it is difficult to draw effective conclusions by directly comparing the data of various indicators. Each element was standardized to obtain the standardized scores of each influencing factor as shown in S2 Table. The correlation matrix of each element was calculated, and the data were subjected to a suitability test for principal component analysis. The result was 0.737 >  0.7, indicating that the data passed the KMO test and that the principal component analysis method could be used to calculate the weights. Based on the variance-explained correlation coefficient matrix, the contribution rate of variable explanation is 92.5% when the principal component is 2. The PCA calculation details can be found in S3–S6 Tables, including KMO and Bartlett’s test, variance explanation, component matrix, and principal component loading.

An integrated scoring model was established to describe the cooling effect based on two principal components, with the calculation method as shown in the following formula. This model describes the cooling effect of the park using seven main indicator elements, which can be used to explain the cooling mechanism of the park as well as to predict the cooling effect of green space parks

F = 0.350x_1_ + 0.348x_2_ + 0.339x_3_ + 0.361x_4_ + 0.327x_5_ + 0.1x_6_ + 0.068x_7_

where x_1_: park area; x_2_: water perimeter; x_3_: park perimeter; x_4_: proportion of tree canopy; x_5_: water area; x_6_: surrounding building plot ratio; x_7_: surrounding building density.

After normalization, the weights of each indicator were calculated to explain the cooling effect. As shown in Table 1, the indicator with the greatest weight is the proportion of tree canopy proportion, with a weight of 19.07%; the indicator with the smallest weight is the surrounding building density, with a weight of 3.60%. It can be seen from this that the inherent nature of the park can explain the vast majority of factors affecting the cooling effect of the green space, including the scale of the green space park and its internal water body (including area, perimeter, etc.), as well as the proportion of tree canopy area within the park. The internal properties of the park can explain about 91.4% of the influencing factors involved in the cooling effect of the green space park, while the external factors (including the surrounding building density and plot ratio) account for 8.86% of the influencing factors.

**Table 1:** Weights of the Indicators.

Indicator	Weight
**Park Area (x**_**1**_)	18.48%
**Water Perimeter (x**_**2**_)	18.39%
**Park Perimeter (x**_**3**_)	17.90%
**Proportion of Tree Canopy (x**_**4**_)	19.07%
**Water Area (x**_**5**_)	17.29%
**Surrounding Building Plot Ratio (x**_**6**_)	5.27%
**Surrounding Building Density (x**_**7**_)	3.60%

### 4.2. Strategies for improving regional thermal environment using park cold islands

With the rapid development of urbanization as the research background, this study focuses on urban issues such as the urban heat island effect and changes in the urban thermal environment. The cooling patterns of urban parks and the factors affecting their cooling effects are explored with the aim of providing quantitative strategic guidance for the planning and design of urban parks or green spaces and offering a new perspective for improving the urban thermal environment. Previous research has analyzed the cooling effect of park cold islands and the underlying mechanisms of influence. It is necessary to identify the areas within Xi’an city that are most in need of green space cold islands in order to propose targeted urban park planning strategies that maximize the role of these islands in improving the urban thermal environment. Using the conclusions obtained from previous research, recommendations are also made regarding the morphological characteristics and distribution locations of the park cold islands themselves.

#### 4.2.1. Demand for cold islands in Xi’an.

It is necessary to clarify in urban planning and design the intended location for green space cold islands to fully leverage the cold island effect of urban parks and thereby improve the thermal environment of the area. Based on the conclusions of the previous manuscript, these areas should meet three conditions: (1) The area should exhibit a significant urban heat island effect. (2) There should be no green spaces or other cold island areas in the vicinity. (3) The establishment of an urban park nearby should produce the greatest cooling effect, maximizing the effectiveness of the investment.

To assess the demand for park cold islands in Xi’an, we conducted a survey on the distribution of heat island areas in this region. High-temperature areas were extracted to form a distribution map of high-temperature areas in Xi’an (red areas); the locations of existing large green spaces or urban parks were also marked (green areas). The distribution map is detailed in S4 Fig.

It can be observed that the high-temperature areas exhibit certain characteristics in their spatial distribution: (1) A large number of high and extremely high-temperature areas are located in the densely populated central urban areas of Xi’an. In addition, there are clearly defined high-temperature areas extending to the east and west of the central urban area. (2) There are two distinct high-temperature areas in the northwest and directly north of the city.

**Fig 16:**
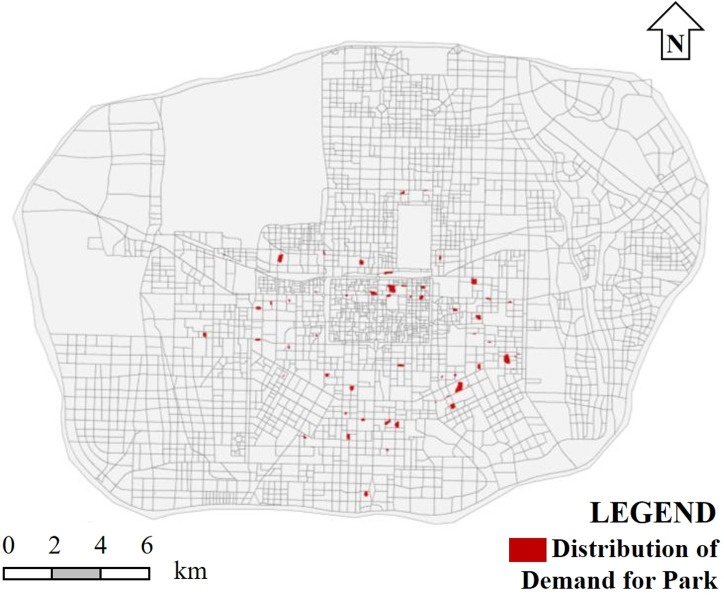
Distribution of demand for park cold islands in Xi’an.

As concluded in previous research, green space cold islands are most effective in cooling areas with a building density of around 0.25–0.3 and a building plot ratio of above 4.0. Establishing green spaces in these areas can maximize the cold island effect of urban parks. Therefore, areas in Xi’an with a building density of 0.25–0.3 and a building plot ratio of 4.0–6.0 were identified. As shown in Fig 16, the indicated areas suffer from severe heat island effects and lack effective parks or green spaces nearby.

#### 4.2.2. Park construction strategy based on thermal environment improvement.

Based on the situation near the heat island area, including the location, whether there is ample surrounding land, and whether there are already parks of scale, the heat island space was divided into two categories, A and B. Heat islands of category A are located in areas with relatively relaxed land use and lack large-scale parks, making it suitable to build green parks of a certain scale around them. Heat islands of category B heat islands are located in areas with relatively tight land use, or where there are already large-scale parks or scenic spots, making them unsuitable for further large-scale park construction. Instead, it is suitable to build smaller activity green spaces or parks in these areas.

**Fig 17:**
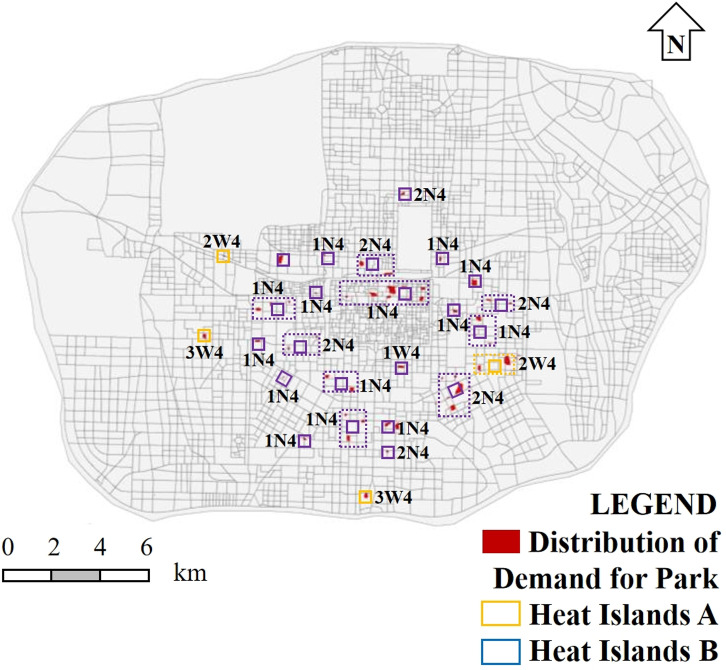
Recommended new categories of parks.

To improve the thermal environment, different types of green cold islands were planned for the above two types of heat islands. According to the analysis in previous research, green spaces with an area of 0.6–0.7 km² can play a good cooling effect. Therefore, in the heat island areas with relatively relaxed land conditions, three types of urban green spaces were planned (0.4–0.7 km²). Type 1 or 2 (as shown in S1 Table) ancillary green spaces to be installed within the land itself are used in areas with tight land use or where there are already parks of a certain scale. For example, green space areas can be increased in commercial plazas to play a cooling role. From the analysis in previous research, it is known that water bodies and tall trees can effectively cool the surrounding built-up areas. When the area ratio of tall trees is more than 60% and the water body area ratio reaches 10%, the cooling effect is good. Therefore, it is suitable to build artificial water features in the green space in type-2 and -3 parks (as shown in Table 1) and increase the number of tall trees as much as possible. Based on this principle, this paper suggests that the following four types of green cold islands can be used to improve the thermal environment (as shown in Fig 17).

Table 2 provides a clearer understanding of the new park models by listing the suitable ranges for specific parameters of four types of proposed parks. Similar types of parks were identified from existing park cases for design reference. It should be noted that, with the exception of Park 1, the tree canopy proportion in other completed parks is generally lower than the recommended values.

**Table 2:** Recommended park categories.

Category	Park Category	Park Area (km^2^)	Water Area Proportion	Tree Canopy Proportion	Reference Park
A	3W4	0.4–0.7	10%–20%	60%^+^	Park 29
A	2W4	0.1–0.4	10%–20%	60%^+^	Park 23
B	2N4	0.1–0.4	0	60%^+^	Park 1
B	1N4	< 0.1	0	60%^+^	Park 5

In summary, in urban park construction and planning, the following steps can be referred to: 1. Obtain urban heat island and existing green space/park areas through satellite data. 2. Acquire the characteristics of surrounding building density and plot ratio in the urban built-up area. 3. Superimpose the results of 1 and 2 to clarify which heat island areas are suitable for improvement with urban parks. 4. Determine the park construction parameters for each area based on the built-up area environment.

### 4.3. Comparison of similar studies

The content of this study leads to similar conclusions to those of existing scientific research in some respects, which can serve as mutual validation to enhance the accuracy of such studies. For example, this is the case in terms of the impact of the tree cover area, park area and perimeter, and water body area and perimeter on the cooling effect of parks. Regarding the influence of building density and floor area ratio on the urban thermal environment, while there are no very similar studies, some studies have also found features that are similar to the characteristics of this study.

Firstly, in the present study, tree cover area was identified as the most important indicator affecting the cooling effect of parks, which is similar to the views in Haiwei’s study [46]. Haiwei also proposed that an increase in tree cover can enhance the cooling effect of trees, especially by increasing the SVF. Secondly, the conclusion that the tree area is positively correlated with the cooling effect of parks can be corroborated by Jiahao’s research, which confirmed that different trees and planting distances have different cooling effects on the thermal environment of semi-outdoor spaces [47]. Thirdly, we found that when the tree area accounts for 50%–60% of the total green space area, the impact of the tree area on the park’s cooling effect is the strongest, which is also similar to Haiwei’s research [46]. Fourthly, regarding the interplay between the park area and perimeter and their cooling effects, we found that the cooling effect is most pronounced when the park area is between 0.6 and 0.7 km² and the perimeter is between 4000 and 4500 m, which aligns with Zhang’s research conclusions [48]. Fifthly, regarding the relationship between the water body area and perimeter and the park’s cooling effect, it was found that when the water body area is between 0.3 and 0.4 km² and the perimeter is about 5000 m, the cooling effect is most pronounced. This conclusion is closely related to the morphology of the water body. However, Zhang’s study in the Wuhan area suggests that for every 10% increase in the proportion of water body area, the park’s cooling intensity gradually increases [49]. Although our research conclusions have different numerical values, the correlation between water bodies and the cooling capacity of green spaces is consistent. Sixthly, the literature mentions that there is a positive correlation between the surrounding building density and the park’s cooling effect, and when the building density is between 0.25 and 0.3, the park’s cold island effect can reach the maximum cooling range. This is in line with the conclusions of previous research [50]. Lastly, we found that this maximum cooling range can also be reached when the building density is between 4.0 and 6.0. This point has not been corroborated by other studies, but Zhang’s other study mentioned that the type, shape, and layout of buildings and the configuration of trees and shrubs affect the degree to which green spaces improve the thermal environment, which also implies that building layout and density have a certain impact on the cooling effect of parks [51].

### 4.4. Limitation

This manuscript utilizes data obtained from remote sensing, which represents only a specific moment in the summer, and the research is conducted based on this momentary data. Establishing long-term monitoring stations and conducting dense, continuous, and on-site monitoring can help to more accurately understand the dynamic patterns of the cooling island effect and cooling effects of green space.

This paper focuses on exploring the cooling patterns from the perspective of the geometric forms of green space cold islands and water bodies, the area of tall trees, and the layout of surrounding buildings. This study has been conducted by treating urban green spaces as two-dimensional environments, future research could also explore the cooling effects of three-dimensional green spaces in terms of plant height, type, and combination methods.

### 4.5. Future research recommendations

This manuscript primarily uses temperature as a quantitative indicator of the thermal environment, employing infrared thermometers to measure surface temperatures. Due to the limitations of the measurement principles of such devices, various environmental conditions such as temperature, humidity, wind speed, and the selection of appropriate surface emissivity can significantly affect the measurement results. Although the accuracy of the equipment used in this study is within 0.1°C, to better obtain accurate and representative data, this paper conducted 20 measurements at each measurement point and used the average value as the representative temperature for that scenario. Based on existing research experience, conducting more than five measurements for each scenario can yield more stable results, thus it can be considered that the results of this study are representative.

However, this does not imply the universality of the related research conclusions. For instance, the study period selected in this paper is sunny, as remote sensing temperature was used, and only on sunny days can more accurate data be derived through computational formulas. However, there is a significant deviation in this conclusion on cloudy days. Therefore, future related research still needs to introduce more computational methods to expand the results. For example, using CFD simulation and other methods to compensate for the poor resolution of fixed-point measurement studies. Furthermore, the spatial planning characteristic of the city in this study is grid-like, so the diffusion of cold islands is better due to the presence of roads. However, this conclusion may not hold for cities with more winding roads, and more research is needed to expand similar conclusions. Additionally, global warming is a major trend in the Earth’s thermal environment, and urban background temperatures will rise year by year. Therefore, how these conclusions will change as urban environmental temperatures further increase is also a topic worth in-depth research in the future.

## 5. Conclusions

Urbanization leads to significant changes in urban surfaces and land use, exacerbating issues like the urban heat island effect, which negatively impacts residents’ health. Green spaces and parks can mitigate this by forming park cold islands, offering cooler and more comfortable environments. Hence, there is a need for a quantitative understanding of these effects to enhance urban living conditions scientifically. This study focuses on urban park cold islands in Xi’an. Field research was conducted on 36 parks using remote sensing data to analyze cooling patterns and mechanisms. The aim was to identify factors affecting park cooling effects and develop urban planning strategies to improve the urban thermal environment and optimize green space design. The main conclusions include the following:

1. It was clarified that the proportion of arbor area is the most important indicator affecting the cooling effect of parks (19.07%), while the indicator with the least influence is the surrounding building density (3.60%).
2. The area and perimeter of the park are related to its cooling effect, and both show a positive correlation. When the area of the green space park is in the range of 0.6–0.7 km² and the perimeter is maintained between 4000 and 4500 m, the most obvious cooling effect can be achieved.
3. The area of arbor trees is positively correlated with the park’s cooling effect, and a significant cooling effect can be achieved when the arbor area accounts for 50%–60% of the total green space area.
4. The area and perimeter of the water body in the park are related to its cooling effect, and both show a positive correlation. When the water body area is in the range of 0.3–0.4 km² and the perimeter is about 5000 m, the most obvious cooling effect can be achieved.
5. The surrounding building density can affect the cooling effect of the park, and there is a positive correlation between the two. When the building density is between 0.25 and 0.3, the park cold island can reach the maximum cooling range.
6. The building plot ratio of surrounding buildings can affect the cooling effect of a green cold island, and there is a positive correlation between the two. When the building density is between 4.0 and 6.0, the park cold island can reach the maximum cooling range.

## Supporting information

S1 Table Green Spaces Park Details. (PDF)

S2 Table Standardized Scores of Each Elements. (PDF)

S3 Table KMO and Bartlett’s Test of Sphericity. (PDF)

S4 Table Variance Explained Table. (PDF)

S5 Table Component Matrix. (PDF)

S6 Table Principal Component Loading. (PDF)

S1 Fig Research location. (TIF)

S2 Fig Characteristics of building density in Xi’an. (TIF)

S3 Fig Characteristics of building plot ratio in Xi’an. (TIF)

S4 Fig Distribution of heat and cold islands in Xi’an. (TIF)
