# Supplementary material for: The impact of urban parks on the thermal environment of built-up areas and an optimization method
Source: PLoS One. 2025 Mar 6;20(3):e0318633. doi: 10.1371/journal.pone.0318633 (PMC11884726; doi:10.1371/journal.pone.0318633)
Supplement: S1 Table — (PDF) [file pone.0318633.s001.pdf]

Number	Name	Scale	Water	Tree
1	Kuangshanlu Greenbelt	2	N	4
2	Olympic Park	3	N	1
3	Mudanyuan Park	1	N	1
4	Wuji Park	1	N	2
5	Xiaoshulin Park	1	N	2
6	Dongyuan Park	1	N	1
7	Dacien Temple Site Park	2	N	2
8	Yujincheng Park	1	N	1
9	Fangzhi Park	1	N	1
10	Second Qin Emperor Mausoleum Site Park	1	N	1
11	Dongneiyuan Park	1	N	1
12	Xi'an Martyrs Cemetery Park	2	N	3
13	Dayanta Park	2	N	3
14	Xi’an Children Park	1	W	1
15	Wenjing Park	1	W	2
16	Geming Park	2	W	2
17	Mutasi Park	1	W	2
18	Xinjiyuan Park	2	W	2
19	Qujiang Cave Ruins Park	1	W	1
20	Labor Park	1	W	1
21	Xiaoyanta Park	2	W	3
22	Hongguang Park	2	W	1
23	Changle Park	2	W	3
24	Yongyang Park	2	W	2
25	Lianhu Park	1	W	1
26	Yunshui Park	1	W	1
27	City Sport Park	2	W	2
28	Fengqing Park	2	W	3
29	Xingqinggong Park	3	W	3
30	Yanming Lake Park	3	W	1
31	Daming Palace Ruins Park	4	W	1
32	Tang Paradise	3	W	2
33	Park Around the City Wall	4	W	3
34	Taohua Lake Park	3	W	2
35	Qujiangchi Site Park	3	W	2
36	Hancheng Lake Scenic Park	4	W	2
