# Supplementary material for: The impact of urban parks on the thermal environment of built-up areas and an optimization method
Source: PLoS One. 2025 Mar 6;20(3):e0318633. doi: 10.1371/journal.pone.0318633 (PMC11884726; doi:10.1371/journal.pone.0318633)
Supplement: S2 Table — (PDF) [file pone.0318633.s002.pdf]

Park Number	Park Area	Park Perimeter	Water Area	Water Perimeter	Tree Canopy Coverage	Surrounding Building Density	Surrounding Building Plot Ratio
Park-1	-0.28882	-0.20241	-0.45534	-0.53913	0.15953	-0.69879	-0.21927
Park-2	0.91525	0.53338	-0.45534	-0.53913	0.22214	-0.70369	-0.91548
Park-3	-0.42260	-0.25713	-0.45534	-0.53913	-0.70330	-0.44340	0.71721
Park-4	-0.60990	-0.70024	-0.45534	-0.53913	-0.70732	0.56303	0.89779
Park-5	-0.63666	-0.80936	-0.45534	-0.53913	-0.71168	1.45887	0.01354
Park-6	-0.58314	-0.62425	-0.45534	-0.53913	-0.70853	0.50773	-0.00884
Park-7	-0.36909	-0.31913	-0.45534	-0.53913	-0.49106	0.25336	-0.46030
Park-8	-0.55639	-0.61053	-0.45534	-0.53913	-0.70234	-1.02956	-0.45433
Park-9	-0.55639	-0.52393	-0.45534	-0.53913	-0.69935	0.31972	-0.55059
Park-10	-0.50287	-0.53460	-0.45534	-0.53913	-0.66432	-0.80837	-0.10809
Park-11	-0.60990	-0.74490	-0.45534	-0.53913	-0.73984	-0.44340	-1.21844
Park-12	-0.15503	-0.19089	-0.45534	-0.53913	0.35309	1.32615	1.28507
Park-13	0.03227	0.19544	-0.45534	-0.53913	0.42452	0.59621	-0.25285
Park-14	-0.58314	-0.64781	-0.44818	-0.49813	-0.62306	2.19986	2.83867
Park-15	-0.44936	-0.42772	-0.43946	-0.38558	-0.41638	0.30866	0.32172
Park-16	-0.36909	-0.29471	-0.40988	-0.39500	-0.24893	1.35933	0.84854
Park-17	-0.42260	0.00537	-0.40866	-0.39657	-0.43300	-1.15122	-0.61178
Park-18	-0.28882	-0.25731	-0.40374	-0.33953	-0.22367	0.57409	0.18591
Park-19	-0.52963	-0.38695	-0.40349	-0.25082	-0.69169	-0.32174	-0.28046
Park-20	-0.44936	-0.43281	-0.40188	-0.32375	-0.34592	0.40532	0.80079
Park-21	-0.28882	0.08836	-0.39210	-0.26905	0.02350	0.72893	0.69781
Park-22	-0.28882	-0.14390	-0.33616	-0.24553	-0.57741	-1.43877	-1.54826
Park-23	-0.18179	-0.06671	-0.32785	-0.06547	0.03704	0.13170	0.44559
Park-24	-0.34233	-0.21787	-0.32132	-0.30368	-0.43147	-1.41665	-1.26695
Park-25	-0.50287	-0.47674	-0.30540	-0.21892	-0.51843	2.15563	2.53347
Park-26	-0.44936	-0.42139	-0.26028	-0.15821	-0.61833	-1.82586	-1.67288
Park-27	0.32659	0.24771	-0.19967	0.30375	0.50442	-0.42128	-0.20137
Park-28	0.03227	0.13566	-0.14679	0.04958	0.40539	0.35290	0.53962
Park-29	0.72795	0.35363	0.36634	0.45294	1.53387	0.19806	0.34784
Park-30	0.43362	0.51444	0.73165	0.77343	-0.32762	-1.27288	-1.34007
Park-32	1.02227	0.50304	1.34805	1.90797	1.50850	0.46349	0.03294
Park-34	1.12930	0.77305	2.45267	1.83892	1.41102	-0.34386	-0.62745
Park-35	1.02227	0.86566	2.60910	1.23270	0.75041	-0.60930	-0.43866
Park-36	4.79501	5.07557	3.61645	4.29960	4.25022	-0.97426	-0.33046
