# Supplementary material for: The impact of urban parks on the thermal environment of built-up areas and an optimization method
Source: PLoS One. 2025 Mar 6;20(3):e0318633. doi: 10.1371/journal.pone.0318633 (PMC11884726; doi:10.1371/journal.pone.0318633)
Supplement: S3 Table — (PDF) [file pone.0318633.s003.pdf]

**Kaiser-Meyer-Olkin Measure of Sampling Adequacy**		.770
**Bartlett's Test of Sphericity**	Approx. Chi-Square	340.947
	Df	21
	Sig.	< 0.001
