# Supplementary material for: The impact of urban parks on the thermal environment of built-up areas and an optimization method
Source: PLoS One. 2025 Mar 6;20(3):e0318633. doi: 10.1371/journal.pone.0318633 (PMC11884726; doi:10.1371/journal.pone.0318633)
Supplement: S4 Table — (PDF) [file pone.0318633.s004.pdf]

Component	Eigenvalue	Variance Percentage	Cumulative %
1	4.720	67.430	67.430
2	1.758	25.108	92.539
3	0.247	3.527	96.066
4	0.151	2.158	98.223
5	0.074	1.050	99.274
6	0.038	0.548	99.822
7	0.012	0.178	100.000
