# Supplementary material for: The impact of urban parks on the thermal environment of built-up areas and an optimization method
Source: PLoS One. 2025 Mar 6;20(3):e0318633. doi: 10.1371/journal.pone.0318633 (PMC11884726; doi:10.1371/journal.pone.0318633)
Supplement: S5 Table — (PDF) [file pone.0318633.s005.pdf]

**Variable**	**Factor**	
	**1**	**2**
**Water Perimeter**	0.979	0.093
**Park Area**	0.966	0.106
**Park Perimeter**	0.961	0.069
**Water Area**	0.933	0.227
**Surrounding Building Plot Ratio**	0.925	0.071
**Tree Canopy Proportion**	-0.255	0.928
**Surrounding Building Density**	-0.334	0.903
