# Supplementary material for: The impact of urban parks on the thermal environment of built-up areas and an optimization method
Source: PLoS One. 2025 Mar 6;20(3):e0318633. doi: 10.1371/journal.pone.0318633 (PMC11884726; doi:10.1371/journal.pone.0318633)
Supplement: S6 Table — (PDF) [file pone.0318633.s006.pdf]

Variable	First Principal Component (F_1)	Second Principal Component (F_2)
**Water Perimeter (x_1)**	0.451	0.070
**Park Area (x_2)**	0.445	0.080
**Park Perimeter (x_3)**	0.442	0.052
**Water Area (x_4)**	0.430	0.171
**Surrounding Building Plot Ratio (x_5)**	0.426	0.054
**Tree Canopy Proportion (x_6)**	-0.117	0.700
**Surrounding Building Density (x_7)**	-0.154	0.681
