# Supplementary figures and images for: The impact of urban parks on the thermal environment of built-up areas and an optimization method
Source: PLoS One. 2025 Mar 6;20(3):e0318633. doi: 10.1371/journal.pone.0318633 (PMC11884726; doi:10.1371/journal.pone.0318633)

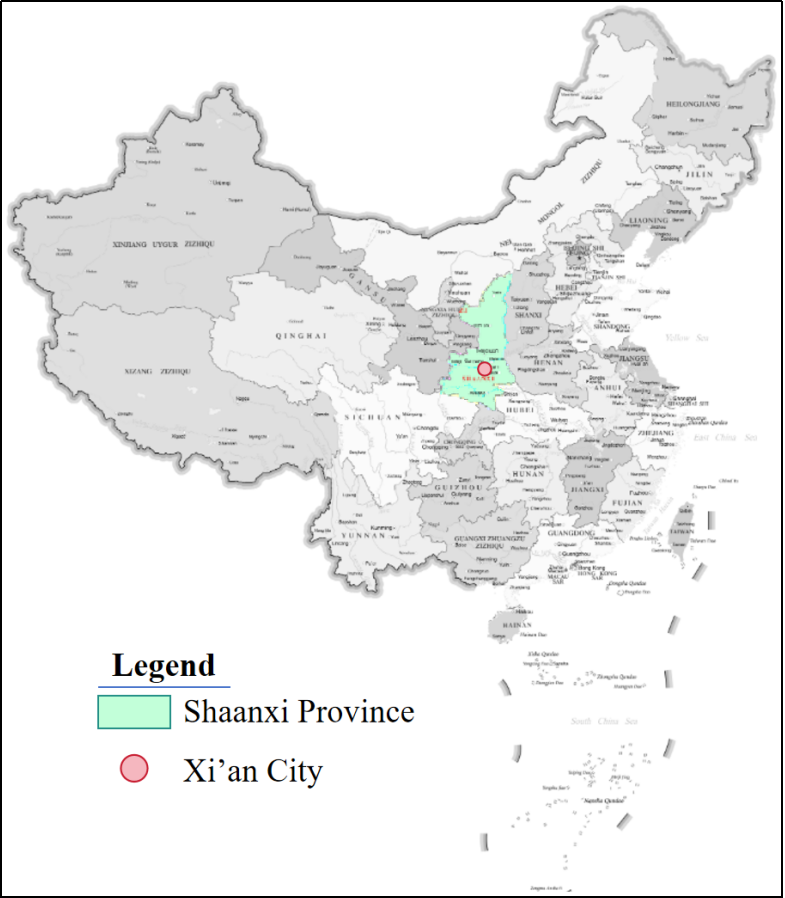

Supplement: S1 Fig — (TIF) [file pone.0318633.s007.tif]

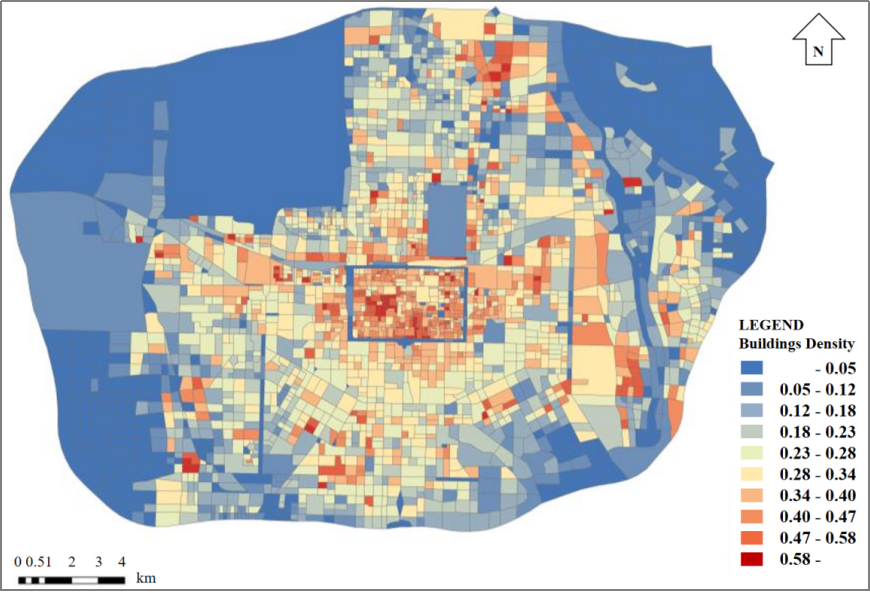

Supplement: S2 Fig — (TIF) [file pone.0318633.s008.tif]

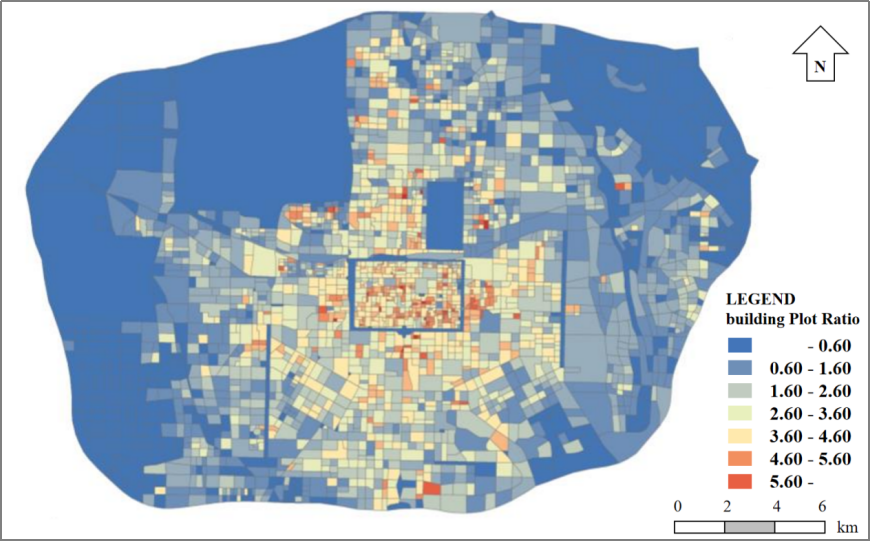

Supplement: S3 Fig — (TIF) [file pone.0318633.s009.tif]

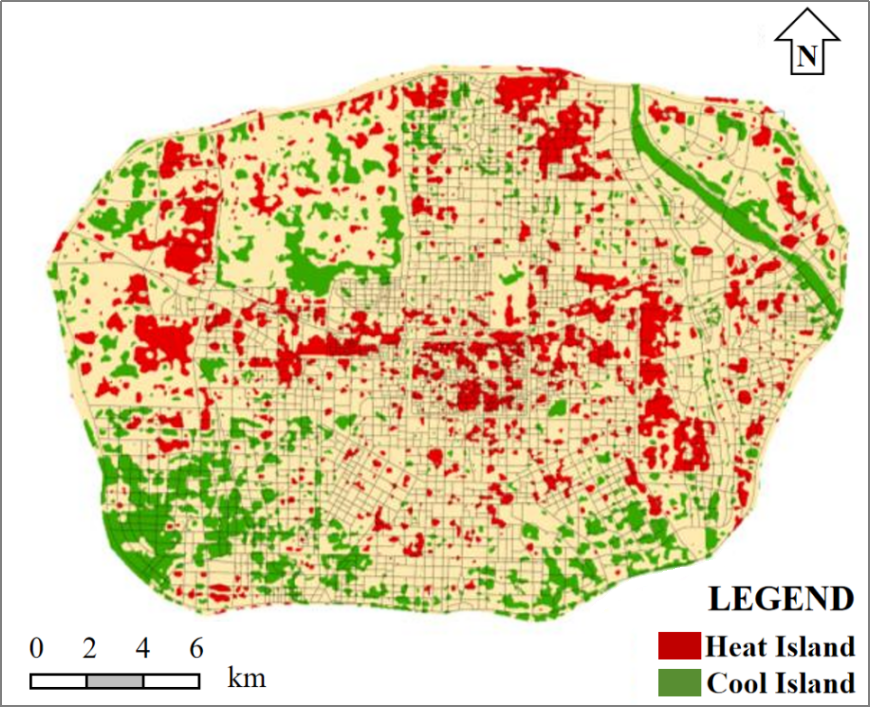

Supplement: S4 Fig — (TIF) [file pone.0318633.s010.tif]
